# Maximum likelihood models and algorithms for gene tree evolution with duplications and losses

**DOI:** 10.1186/1471-2105-12-S1-S15

**Published:** 2011-02-15

**Authors:** Pawel Górecki, Gordon J Burleigh, Oliver Eulenstein

**Affiliations:** 1 Institute of Informatics, Warsaw University, Warsaw, 02-097, Poland; 2 Department of Biology, University of Florida, Gainesville, 32611, USA; 3 Department of Computer Science, Iowa State University, Ames, 50011, USA

## Abstract

**Background:**

The abundance of new genomic data provides the opportunity to map the location of gene duplication and loss events on a species phylogeny. The first methods for mapping gene duplications and losses were based on a parsimony criterion, finding the mapping that minimizes the number of duplication and loss events. Probabilistic modeling of gene duplication and loss is relatively new and has largely focused on birth-death processes.

**Results:**

We introduce a new maximum likelihood model that estimates the speciation and gene duplication and loss events in a gene tree within a species tree with branch lengths. We also provide an, in practice, efficient algorithm that computes optimal evolutionary scenarios for this model. We implemented the algorithm in the program DrML and verified its performance with empirical and simulated data.

**Conclusions:**

In test data sets, DrML finds optimal gene duplication and loss scenarios within minutes, even when the gene trees contain sequences from several hundred species. In many cases, these optimal scenarios differ from the lca-mapping that results from a parsimony gene tree reconciliation. Thus, DrML provides a new, practical statistical framework on which to study gene duplication.

## Background

One of the fundamental problems in evolutionary biology is to determine the genomic mechanisms that generate phenotypic and species diversity. Gene duplications play a critical role in acquiring new gene functions and, consequently, adaptive innovations (e.g., [1-3]). Recent surveys of genomic data have revealed tremendous variation in gene content and copy number across species (e.g., [4,5]). Scientists are now challenged to place this variation in an evolutionary context, that is, to determine where in evolutionary history the gene duplications took place. This is the first step in linking the genomic changes to phenotypic changes or shifts in diversification rates.

Gene tree–species tree reconciliation provides a direct approach to infer the patterns and processes of gene duplication and loss within the evolutionary history of species. Evolutionary processes such as gene duplication and loss, lateral transfer, recombination, and incomplete lineage sorting (deep coalescence) create incongruence between the gene trees and the phylogenies of species in which the genes evolve (e.g., [6]). Gene tree–species tree reconciliation problems seek to infer and map the evolutionary events that caused the incongruence. In this paper, we introduce a novel, and in practice, efficiently computable maximum likelihood approach for reconciling gene tree and species tree topologies based on gene duplications and losses, and we demonstrate its performance using simulated and empirical data sets.

### Related work

**Figure 1:**
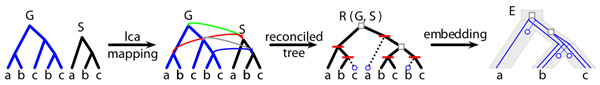
**LCA-reconciliation**. Example of a reconciliation based on a parsimony between the gene tree G and the species tree *S*. The reconciled tree defines the evolutionary scenario with the minimal number of gene duplications and gene losses. *R*(*G*, *S*) has two gene duplications, denoted by squares, and three gene losses, denoted by circles and dot lines. The red horizontal bars in R(G, S) denote speciation events.

The first model to reconcile gene trees with species trees was the gene duplication model, which was introduced by Goodman et al. [7] (see also [8]). In the gene duplication model, a gene tree can be embedded into a species tree through least common ancestor mapping (lca-mapping), which maps every node in the gene tree to the most recent node in the species tree that could have contained the ancestral gene (Figure 1). A node in the gene tree represents a duplication if it has a child with the same lca-mapping. This mapping also represents the most recent possible location of the gene duplication and/or loss, and it represents the most parsimonious reconciliation hypothesis in terms of gene duplications and losses. In other words, the lca-mapping implies the fewest number of gene duplications, or duplications and losses needed to reconcile the gene trees with the species tree.

Minimizing the number of gene duplications and losses through lca-mapping appears to produce relatively accurate mappings of gene duplications and losses when the rates of gene duplication and loss are slow [9,10], and it can be computed in linear time [11,12]. Moreover the parsimony criterion has been used effectively in phylogenetic inference, in which, given a collection of gene trees, the goal is to find the species tree that minimizes the number of duplications or duplications and losses (e.g., [13-18]). However, there are usually many other possible locations of duplication and loss events besides the ones implied by the lca-mapping (e.g., [10]), and some of the most biologically interesting genes, such as the MHC gene family or the olfactory receptor genes, have high rates of duplication and loss. Furthermore, the parsimony criterion fails to consider evolutionary time, which is typically represented by the branch lengths on the species tree. For example, if a duplication could have occurred on two branches, one representing one million years and the other representing 100 million years, all else being equal, it would be much more likely that the duplication occurred during the one hundred million year interval. Yet, a parsimony model would not consider this information. Finally, it is difficult to incorporate the parsimony criterion into a rigorous statistical framework to examine evolutionary hypotheses associated with gene duplication.

There has been much recent interest in likelihood-based approaches for reconciling gene trees and species trees, much of which has focused on coalescence models to describe incomplete lineage sorting (e.g., [19,20]). Probabilistic modeling of gene duplication and loss is relatively new and has largely focused on birth-death processes [9,21-24]. Although these approaches and models are promising, they represent a narrow range of potential models, and are computationally complex.

### Contributions

We describe a novel, efficiently computable maximum-likelihood model-based approach for gene tree reconciliation. Our initial model infers evolutionary scenarios from a gene tree and a species tree with branch lengths, which may represent the time between neighboring speciation events. Our model allows the use of almost any discrete distribution to model gene duplications throughout the species tree. More precisely, we assume that for every branch of the species tree there is a given discrete distribution, which is parameterized by its length. The branch length defines the probability of having *n* gene duplications on this branch. Based on this model, we define the following *maximum likelihood problem*: given a gene tree and a species tree, find the gene tree reconciliation with the maximum likelihood.

To address and ultimately solve this complex problem, we model formally the notion of an evolutionary scenario (the evolution of a gene tree) and prove its equivalence to the model of DLS-trees [25]. Despite the complexity of the possible scenarios of gene duplication and loss, we provide an in practice efficient algorithm for the maximum likelihood problem based on dynamic programming, and use it to reconstruct the optimal placement of gene duplications and optimal evolutionary scenarios. We show that the dynamic programming approach can be efficiently applied in almost all instances (> 99.7% of our simulation experiments) of the maximum likelihood problem. Additionally, we provide a branch and bound solution for the few remaining instances that are not solved by the dynamic programming approach.

We developed DrML, a prototype implementation of the algorithms in Python, and demonstrate its performance on empirical and simulated data. DrML identifies the maximum likelihood gene tree reconciliation in a few minutes on problems with several hundreds of species and gene sequences.

## Methods

### Basic notation and preliminaries

A *gene tree* is a rooted, binary, and directed tree whose leaves are labelled by the species names. A *species tree* is a gene tree whose leaves are uniquely labelled. Let *T* be a gene tree. For a node *v* ∈ *T* we denote by *T*(*v*) the subtree of *T* that is rooted at *v*. By root(*T*) we denote the root of *T*. A node is called *internal* if it has two children. By *L*(*T*) we denote the set of leaves of *T*, and by *L*(*T*) we denote the set of leaf labels of *T*. In this paper we assume that *L*(*G*) ⊆ *L*(*S*) for a gene tree *G* and a species tree *S*.

We define ≤ to be the partial order on the set of nodes of *T*, where *x* ≤ *y* if *y* is a node on the path between the root of *T* and *x*. The *least common ancestor* of a non-empty subset of nodes *X* ⊆ *T*, denoted by lca(*X*), is the unique smallest upper bound of *X* under ≤. *Mapping* is a function m from the nodes of gene tree *G* into the nodes of a species tree *S* that preserves the leaf labels, and satisfies: **(1)** for all *u, v* ∈ *G*, if *u* ≤ *v* then *m*(*u*) ≤ *m*(*v*), and **(2)** for all *g* ∈ *G*, lca(*m*(*L*(*G*(*g*)))) ≤ *m*(*g*). The special case of a mapping, where the equality holds in the second condition, is called *lca-mapping* and denoted by m*. An internal node *g* ∈ *G* is called *lca-speciation* if and only if its children are not mapped into m*(*g*).

#### Modeling evolutionary scenarios

Informally an evolutionary scenario is equivalent to an embedding of a gene tree into a species tree (see Figure 1). A gene can be lost or duplicated into new copies by a gene duplication or speciation event. Both speciation and duplication create two copies of a gene. However, the duplication event occurs in one species and produces two copies of the gene (called paralogs) in the same species, while the speciation creates two new species, each with a single copy of the gene (called orthologs). In order to model the evolutionary scenario, we first need to state whether an internal node of the gene tree represents a speciation or a duplication event. Additionally, we have to “locate” these events from the gene tree in the species tree. The locations are described by mappings.

Next, we present the evolutionary scenario called *reconciliation*.

**Definition 1** (reconciliation). *A pair R =* 〈m, Σ〉*, where m is a mapping and* Σ *is a set of nodes from G, is called* reconciliation *if G is lca-speciation and m*(*g*) = m*(*g*)*, for each g* ∈ Σ*. The elements of* Σ *are called* speciations *(in R)*.

Let *R =* 〈m, Σ〉 be a reconciliation. Note that the speciations are internal in *G*. It should be clear from the introduction that other internal nodes of *G* will be called *duplications*. We define dup_R_(*s*) to be the number of duplication nodes for which the mapping m is equal to *s*. Similarly, we define spec_R_(*s*) to be the number of speciation nodes. By R* we denote the *lca-reconciliation* 〈m*, Σ*〉, where Σ* is the set of all lca-speciations.

A reconciliation can be used to model an evolutionary scenario that does not contain instances of the following: (1) a duplication and an immediate loss of one of the descendant copies, or (2) a gene which is lost after a speciation event in all new formed species. Such cases cannot be detected because there is no existing evidence of the loss events.

**Figure 2:**
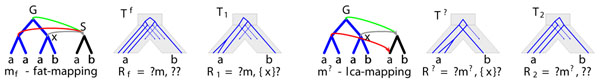
**Reconciliations.** Reconciliations for the gene tree *G* and the species tree *S*. Only two mappings from *G* to *S* exist: the lca-mapping m*, and the mapping *m_f_*. There are 4 possible reconciliations: R*, *R*_1_, *R*_2_ and *R_f_*. The diagram presents the embeddings of *G*, *S*, in which T*, *T*_1_, *T*_2_ and *T_f_* corresponds to R*, *R*_1_, *R*_2_ and *R_f_*, respectively. Only node *x* is the lca-speciation.

It is not difficult to see that there is one-to-one correspondence between reconciliations and semi-normal DLS-trees where DLS-tree is a formal model of evolutionary scenario in the duplication-loss model introduced in [25]. Semi-normal DLS-trees cover the most important and representative part of the scenarios space. The example of reconciliations is presented in Figure 2. In general, the number of possible reconciliations is exponential in the size of gene and species trees. Górecki et al. [25] provide more details on properties of evolutionary scenarios and the DLS-trees.

#### Model and problem

From now on we will use an extended notion of the species tree with branch lengths. For a node *s* ∈ *S* we denote by |*s*| the branch length associated with *s*. Informally, |*s*| can be treated like a branch length of the edge connecting *s* with the parent of *s*. Note, that the root has no edge of this property. However, the notion of the tree could be easily extended to having the root edge.

Let *P*(*τ, d*|λ) denote the probability that *d* duplications occurred during the time period *τ* under the assumption of a constant duplication rate λ. Without loss of generality, we use the Poisson distribution: . The likelihood of a given reconciliation *R* of a species tree *S* and a gene tree *G* is defined by:

*L*(*S*, *G, R*) *=* Π*_s_*_∈_*_S_P*(|*s*|, dup*_R_*(*s*)|λ). (1)

**Definition 2** (optimal reconciliation). *Given a species tree S with branch lengths and a gene tree G, we call the reconciliation R* optimal *if it maximizes the likelihood L*(*S*, *G, R) in the set of all reconciliations of S and G*.

**Problem 1** (Maximum Likelihood Estimation -MLE). *Instance: A species tree S with branch lengths and a gene tree G. Find: The likelihood of an optimal reconciliation*.

**Problem 2** (Duplication-Speciation Setting - DSS). *Instance: A species tree S with branch lengths and a gene tree G. Find: For each s ∈ S find* dup*_R_*(*s*) *and* spec*_R_*(*s*) *such that R is an optimal reconciliation*.

**Problem 3** (Maximum Likelihood Scenario- MLS). *Instance: A species tree S with branch lengths and a gene tree G. Find: An optimal reconciliation*.

### Solutions

We present a dynamic programming (DP) formula for solving the majority of instances of the MLE problem (a complete solution is presented after introduction of *hard instances)*. This formula can be naturally extended to reconstruct duplication-speciation settings (the DSS problem). First we introduce necessary definitions related to reconciliations. For a mapping *m* from G into *S*, let *φ*(*m*,*s*) be the number of internal nodes of *G* that are mapped into *S*(*s*) under *m* (formally *φ*(*m, s*) :*=* |{*g*: *g* is internal in *G* such that *m*(*g*) ≤ *s*}|). Now we define a notion of *acceptable triplet*, which is used to define reconciliations in the algorithm.

**Definition 3.***Let G be a gene tree and S be a species tree. A triplet* 〈*σ, κ*_1_*,κ*_2_〉 *is called* acceptable *for a node s* ∈ *S and its children s_1_ and s_2_ iff there exists a reconciliation* 〈*m*, Σ〉 *such that under mthe following conditions are satisfied: (i) G has exactly σ speciation nodes mapped into s, and (ii) the number of internal nodes of G, which are mapped into S*(*s_k_*)*, equals* κ_k_*for k =* 1,2*. The set of all acceptable triplets for given nodes s, s_1_ and s_2_ is denoted by Acc(s, s*_1_*, s*_2_).

#### Algorithm DP1

For a given *S* and *G* return *M*(root(*S*), |*L*(*G*)| –1) where *M:S* x ℕ → ℝ ∪{–∞} is defined as follows, for *s* ∈ *S* and *κ =* 0 ... *φ*(m*, s) (and *M*(*s*,*κ*) := –∞ in other cases): (i) if *s* is a leaf then: *M*(*s, κ*) :*= P*(|*s*|*, κ*|*λ*), (ii) if *s* is an internal node with two children *s*_1_ and s_2_ then *M*(*s,κ*) equals:(2)

where *p*(*s*,*δ*) denotes the probability of *δ* duplications on branch that terminates in *s* (for example, *P*(|*s*|,*δ*|λ)), *δ =* 0 ... *κ, σ =* 0 ... spec_R__*_(s), *κ = κ*_1_+*κ*_2_+*σ* + *δ* and 〈σ, *κ*_1_, κ_2_〉 ∈ Acc(s, *s*_1_, s_2_).

Algorithm DP1 describes a DP formula for the MLE problem, which we detail in the following. Consider embeddings that are located in a subtree *S*(*s*), for some reconciliations. Informally, *M*(*s,κ*) denotes the maximal likelihood value in the set of all reconciliations under the following conditions: (i) only the embedding (a part of reconciled tree) located in *S*(*s*) is evaluated for the log-likelihood, (ii) *κ* is the total number of duplication and speciation nodes, which are located in this part of embedding, (iii) *δ* is the number of duplication nodes associated with *s*, and (iv) *σ* is the number of speciation nodes associated with *s*.

As mentioned earlier, the DP formula reconstructs the settings of duplication and speciation nodes, or the numbers of these events associated with the nodes of the species tree (see DSS problem). Formally, a *DS setting*, or shorter a *setting*, is defined as a pair of two functions dup, spec: *S* → ℕ, called *distribution of duplications* and *distribution of speciations*, respectively. The distributions of duplications can be reconstructed for internal nodes of *S* from values of variable *δ* in formula (2), and for leaves from *κ*. Similarly, we can use variable *σ* (or 0 in case of leaves) for reconstructing the distribution of speciations. We call a setting 〈dup, spec〉 *valid (for G and S)* if there exists a reconciliation *R* (of G and *S)* such that dup = dup*_R_* and spec = spec*_R_*. The following theorem states an appealing property of the MLE problem.

**Theorem 1.***If at least one of the DS settings reconstructed from Algorithm DP1 is valid then L*(*S*, *G, R) is equal to M*(root(*S*)**,** |*L*(*G*)| –1)*, where R is an optimal reconciliation*.

In general, Algorithm DP1 may result in values that are larger than the likelihood of the optimal reconciliation. However, we show later that such instances, which we call *hard*, are extremely rare and occurring in only 0.1 – 0.4% of random gene tree simulations. The general solution is described later in the paragraph about hard instances. Algorithm DP1 solves a different problem than the DP algorithm presented in [21]. Arvestad et al. [21] present a solution for computing the likelihood only when the reconciliation is given. In contrast, our approach has the following properties: (1) we maximize the likelihood over all reconciliations (Algorithm DP1) requires a gene tree and a species tree with branch lengths only), (2) we use a flexible model of gene duplication based on aggregating duplications on the species tree edges, which differs from a birth-death process.

#### Reconstruction reconciliation – MLS problem

We briefly introduce the general idea of our algorithm for reconstructing a reconciliation from a setting 〈dup, spec〉**.** This algorithm is enumerating all variants with an additional filtering, which is given by some constraints depending on the setting and the properties of the scenarios. This approach requires exponential time in the worst case. However, as we demonstrate, it can be successfully applied to the majority of cases.

Algorithm DSR, presented below, first allocates speciation nodes (Σ) and then duplications nodes (*D*) with mappings. However, before reconstructing mappings of the duplication nodes some of the speciation configurations can be rejected. We now briefly explain a filter process used in the 2nd step of the main loop. Consider internal nodes *g* and *g'* in *G* such that *g< g'* and *g'* ∈ Σ. Then *m*(*g*) <*m*(*g'*), where *m* is the mapping. In other words, the mapping of *G* is ‘locked’ by the mapping of *g'*. Let α(Σ, s) denote the number of nodes in *G* that are locked by s ∈ *S* (formally *α*(*s*, Σ) = |{*g* ∉ *L*(*G*): there exists *g'* ∈ Σ such that *G* <*g'* and m*(*g'*) <*s*}|).

Algorithm DSR is utilized to determine whether a given setting is valid and to reconstruct all reconciliations (with a straightforward modification).

#### Algorithm DSR

Input: Gene tree *G*, species tree *S* and DS setting 〈dup, spec〉. Output: A reconciliation *R* of *G* and *S* such that 〈dup, spec〉 = 〈dup*_R_*, spec*_R_*〉 (if exists). For each subset Σ of Σ* that satisfies the distribution spec set members of Σ to be speciations and inherit their mappings from the lca-reconcilation. Execute 1-3 for each Σ:

1. Let *D* be the set of all internal nodes of *G* that are not in Σ. Set all members of *D* to be duplications.

2. Reject Σ if there exists *s* ∈ *S* such that (i) α(*s*, Σ) >*∑_a_*_<_*_s_* spec(*a*) + dup(*a*) (too many locked below s) or (ii) φ(m*, s) – *α*(*s*, Σ) – spec(*s*) < dup(*s*) (too few for s-duplications).

3. Allocate mappings for the nodes in *D* according to the distribution dup. If the allocation was found return 〈*m*, Σ〉 where *m* is the reconstructed mapping.

#### Acceptable configuration

First, we explain: why we do not enumerate all possible triples of 〈σ, *κ*_1_, *κ*_2_〉 under the conditions given in the formula (2) instead of constraining them to Acc.

As an example, consider the gene tree ((*a*, *b*), ((*a*, (*a*, *a*))*,b*)) and the species tree *S =* (*a*, *b*). The lca reconciliation consists of: 1 duplication and 2 speciation nodes associated with the root of *S*. Observe that there is no reconciliation where the root has 2 duplications and 2 speciation nodes. Similarly, there is no reconciliation with 4 duplications and 1 speciation node in the root. However, without the Acc constraint the DP formula could result in a likelihood computed for one of these invalid duplication-speciation settings. Consequently, only reconciliation based configurations are required to increase significantly the number of valid settings reconstructed from the DP formula.

For the previous example and nodes *ab, a* and *b* we have the following acceptable triplets: 〈2,2,0〉 (lca-reconciliation), 〈1,2,0〉, 〈1,1, 0〉, 〈1,0,0〉, 〈0, 2,0〉, 〈0,1,0〉 and 〈0,0,0〉.

To solve the general problem of acceptability we formulate a problem SeqPair.

**Problem 4** (SeqPair).

*Instance: Integers α* ≥ 0*, β* ≥ 0 *and a sequence A of pairs of nonnegative integers:* 〈*α*_1_, *β*_1_〉, ..., 〈*α*_s_, *β_s_*〉. *Find: The length**of the longest subsequence of A satisfying**and*.

SeqPair can be solved with with the DP formula similar to the DP solution of the Knapsack problem [26]. However, in our case the algorithm is polynomial due to the constraint . This inequality can be deduced from the applications of SeqPair to sets of size s of ≤-incomparable lca-speciations from *G*. With this constraint the algorithm completes in at most  steps.

Now we show how to utilize the solution of SeqPair. For a given reconciliation, *s* ∈ *S* and its children *s*_1_ and s_2_ let *G_s_* be the maximal set of maximal disjoint subtrees of *G* such that for *T* ∈ *G_s_* the nodes of *T* are lca-mapped (that is, under lca-mapping) into nodes of *S*(s*_i_*) for *i =* 1 or *i =* 2. Such *T* is called an *s_i_*-tree. There are spec_R*_(*s*) speciation nodes in *G* lca-mapped into *s*. For each such node *g*, the two subtrees rooted at children of G are elements of *G_s_*, while one of them is an s_1_-tree and the second is an *s*_2_-tree. Such subtrees will be called *dual*. Note that not all trees in *G_s_* are dual. Such trees are called *free*.

In our example of the gene tree ((*a, b*), ((*a,* (*a, a*))*, b*))*,* if *s* is the root of (*a, b*) then *G_s_* contains 2 pairs of dual subtrees.

**Lemma 1.***Let G_s_ contain only dual trees:**where**are**are dual and**is an s_j_-tree. Let  be the number of internal nodes of**. Then* 〈*σ,κ*_1_*,κ*_2_〉 ∈ *Acc*(*s, s*_1_*, s*_2_) *for j =* 1,2 *and σ* ∈ {0, ...,σ*} where σ* is the solution of the Seq-Pair problem for κ_1_*,* κ_2_*and a sequence of pairs:*.

In the example:   (with two internal nodes) and . Thus the sequence of pairs is: 〈0, 0〉, 〈2,0〉 and the solutions are: σ* = 1 if *α* ∈ {0,1}, *β* = 0 and σ* = 2 if *α* = 2, *β* = 0. From Lemma 1 we can easily reconstruct all seven acceptable triplets. The next lemma solves a general case.

**Lemma 2.***For j =* 1,2 *let**be the number of internal nodes of all free s_j_-trees in G_s_ and for i* > 0 *let**be defined like in the previous lemma (for dual trees). Then* 〈*σ,κ*_1_*,κ*_2_〉 ∈ *Acc*(*s, s*_1_*, s*_2_) *iff**for j =* 1,2 *and σ* ∈ {0, .. ., σ*} *and where for some**and*, σ* is the solution of the SeqPair problem for κ_1_*–p,* κ_2_ – q*, and a sequence of pairs:*.

Consider a new example: *G=* (((*a*, *a*)*,* (*b*, *b*))*,* ((*b*, *b*), *b*)) and a species tree (*a*, *b*). There is one free tree for *G_root_*(*g*): *T =* ((*b*, *b*), *b*) and one pair of dual trees: . In our case:  and . Thus, σ* = 1 for *κ*_1_*=* 1 and κ_2_ ∈ {1, 2, 3} and σ* = 0 otherwise.

We analyze the complexity of a single Acc query. Reconstruction of the dual and free trees requires lca-mapping and can be easily computed only once in linear time *O*(|*G*| *+* |*S*|) [*27*]. From Lemma 2 we need at most |*L*(*G*)|^2^ SeqPair queries to solve a single Acc query. All SeqPair queries share the same sequence of pairs. It can be shown that the queries can be answered in constant time after a single preprocessing that construct the DP-array (see the solution of SeqPair). Thus, a single Acc query can be solved in at most  steps.

Finally, we analyze the complexity of Algorithm DP1. Computing *Acc* for all nodes has time complexity *O*(|*S*||*G*|^3^). If the *Acc* queries are cached then the time complexity of Algorithm DP1 is *O*(|*S*||*G*|^4^) from (2). The space complexity is determined by: (i) the DP formula (*M*): |*S*||*G*|, (ii) the dictionary of *Acc* queries for a given node *s:* |*G*|^2^ (note that only maximal *σ* from query should be stored) and (iii) the *SeqPair* DP-array: |*G*|*^3^*.

#### Hard instances

There are *hard* instances of the MLE problem that cannot be resolved with the DP formula (2) (discussed after Theorem 1). Here, we solve the general MLE problem (that also covers the hard instances) by developing a branch and bound algorithm with recursive applications of a DP formula, which is similar to the previous one. First we describe the DP formula that computes the likelihood in constrained sets of reconciliations. Then, we introduce the branch and bound algorithm.

#### DP with constraints for MLE

We begin with the definition of constrained reconciliations. The constraint is defined by two sets of internal nodes of *G*: *F* ⊆ Σ* and *L*. The elements of *F* and *L* are called *raised* and *locked,* respectively. By *Rec*(*F, L*) we denote the set of all reconciliations *R =* 〈*m,* Σ〉 such that (i) m*|*_L_ = m*|*_L_* (locked node remain locked), (ii) *L*∩Σ* = *L*∩Σ (locked lca-speciations remain speciations), (iii) Σ and *F* are disjoint (raised lca-speciations must be duplication in *R*). Thus, *Rec*(*F, L*) contains reconciliations such that the properties of locked nodes (like mappings, being speciation/duplication) are preserved while the raised lca-speciation nodes are duplications. Under this definition, the set of locked nodes can be extended by adding further nodes which share the same “locking” properties. Without loss of generality we assume that *L* is closed under the following conditions: (i) if *g* ∈ *L* ∩ Σ, *g* → *c* ∉ Σ and *m*(*g*) *→ m*(*c*) then *c* ∈ *L,* (ii) if *G* ∈ *L \* Σ and *g→ c* ∈ Σ then *c* ∈ ∈ *L,* where → denotes a child relation in the tree; that is, *a* → *b* iff *b* is a child of *a*. The closure operation will be denoted by .

#### Algorithm DP2

For a given *S ,G, F* ⊆ Σ* and *L* return *M_F,L_*(root(*S*)*,* |*L*(*G*)| *–* 1) where *M_F,L_*: *S* × ℝ → ℕ∪{– ∞} is defined as follows, for *s* ∈ *S* and *κ =* λ(s)... *φ*(m*, s) (and *M_F,L_*(*s, κ*) := –∞ in other cases): (i) if *s* is a leaf then: *M_F,L_*(*s,κ*) := *P*(|*s*|, κ|λ), (ii) if *s* is an internal node with two children *s*_1_ and *s*_2_ then *M_F,L_*(*s,κ*) equals:

where *δ =* |*L_s_* \ Σ*|... *κ – λ*(*s*_1_) – λ(*s*_2_) – |*L_s_* ∩ Σ*|, *σ =* |*L_s_* ∩ Σ* |... spec_R*_ (s) – |Σ* \ F|, κ = *κ*_1_*+* κ_2_ + *σ + δ, p*(*s,δ*) is defined in Alg. DP1, 〈*σ,κ*_1_*,κ*_2_〉 ∈ Acc(*s*, *s*_1_, *s*_2_, *F*, *L),* where Acc(s, *s*_1_*,s*_2_, *F, L)* is the set of acceptable triples for *s* in the set of reconciliations *Rec*(*F,L*)*, L_s_ = L* ∩ m*^–^^1^(*s*) is the set of locked nodes whose lca-mapping is s, and λ (*s*) = |∪_g∈__L,m*__(_*_g_*_)≤_*_s_ G*(*g*) \ *L*(*G*)| is the number of *s-blocked nodes,* that is, internal nodes whose parent is locked and lca-mapped into *S*(*s*).

Algorithm DP2 describes the constrained variant of DP1, where the reconciliations are limited by raised and locked nodes. Computing acceptable triplets in this version is similar to the schema given by Lemma 2 and therefore omitted for brevity. However, it is more complex due to locked and raised nodes. Formally, formulating an analogous lemma for the constrained case the following differences must be adopted: (i) dual trees for locked speciations are omitted, (ii) dual trees for raised lca-speciations become free trees, and (iii) all *s*_1_ and s_2_-blocked nodes are excluded from all free and dual trees. A formal presentation of the lemma is omitted for brevity. Note that Algorithm DP2 has the same time complexity as Algorithm DP1.

#### Branch and bound algorithm for MLE

The concept of this algorithm is based on the branch and bound schema, whis is adequately adapted for the constrained DP. We assume that *extDP*(*F, L*) denotes Algorithm DP2 with the validation of settings (see previous sections), that is, it returns either the maximum likelihood estimation if there exists a valid setting (resolving case) or returns –∞ otherwise (non-resolving). In a single step of the BB solution there are defined sets of locked *L* and raised *F* lca-speciations. We take a non-raised and non-locked lca-speciation *s* and compute  and . Depending on four possible cases (resolving, non-resolving) we either return a value or recursively apply BB procedure with modified *F* and *L*. Note that this approach has an exponential runtime. We omit technical details for brevity.

## Results

### Algorithm implementation

The described programs were implemented as a prototype Python program, called DrML (available at http://bioputer.mimuw.edu.pl/~gorecki/drml/). Specifically, DrML takes a gene tree topology and its corresponding species tree with branch lengths and identify the optimal evolutionary scenarios (scenarios with the highest likelihood) based on the duplication-loss model. Although it is possible to use a broad variety of different distributions to describe the placement of gene duplications events with our algorithms, in DrML we use a Poisson distribution. This assumes a constant rate of duplication throughout the tree, although again, this assumption can be removed by using our algorithm with other distributions. Further detail about the implementation can be found on the DrML web page.

### Simulated data analysis

We first tested the performance of DrML with randomly generated species and gene trees. For each *n =* 10,14,..., 198, we randomly generated 6000 species trees with *n* leaves. The branch lengths of the species trees were sampled from a uniform distribution across the interval [1...20]. For each species tree, we also generated a random gene tree topology with n ï€ª1.25 leaves. Tests of “DP time (all)” were performed with 100 replicate pairs of random species and gene trees.

### Empirical analysis

We also examined the performance of DrML using a gene tree from the TreeFam database [28], specifically accession TF105503 (RING-box protein 1) from TreeFam 7.0. We used a species tree generated from TreeFam, with the branch lengths obtained from diversification dates in the TreeTime database [29]. To root the gene trees, we first identified all most parsimonious rootings (the rootings that minimize the number of duplications) using Urec [30]. All parsimony rootings have the same DS settings, and the corresponding optimal lca-reconciliations are almost identical [31]. Thus, we arbitrary choose one of the parsimonious rootings. For the analysis, we set the duplication rate (λ) to 0.005 following the estimated rate of gene duplication and loss in the vertebrate genome by Cotton and Page [32].

## Discussion

### Simulation analysis

**Figure 3:**
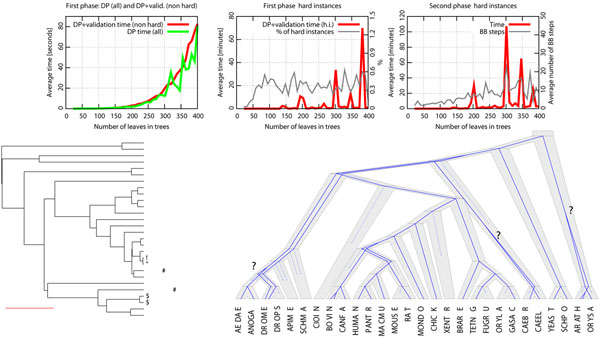
**Experimental results.** [Top part] Performance of DrML prototype. The left diagram presents the performance of DP Algorithm DP1 and DP with validation. The middle diagram shows the percentage of hard instances with the average validation time. The right diagram depicts the average time of computations for hard instances and the average number of branch and bound steps. [Bottom part] An optimal reconciliation for the empirical tree. Arrows denote the duplications that are not present in the lca-reconciliation.

DrML performs well with the simulated data sets even for large trees with almost 400 leaves in the trees; the algorithm still finished in less than 90 seconds on average. The hard instances occurred in only 0.3% of the simulated data sets (Figure 3). In the middle diagram of Figure 3, the peaks in time represent the exponential implementation of MLR problem. This situation may occur when some special cases of hard instances have dense composition of possible duplications. However, among the nearly 300,000 randomly generated data sets, this occurred only 3 times.

### Empirical analysis

For our empirical example, DrML found an optimal reconciliation (logML = –29.412) with one optimal DS setting and one reconciliation. The optimal reconciliation contained 3 duplications that are not found in the lca-reconciliation. These duplications were found on long branches in the species tree, suggesting, as we would expect, that the longer branches are more likely to contain duplications in likelihood reconciliations.

## Conclusions

Our algorithms provide, in practice, a highly efficient and exact approach to infer maximum likelihood based gene tree reconciliations for a novel set of models. In contrast to parsimony based gene tree reconciliations [7,8], these algorithms can incorporate evolutionary time (species tree branch lengths) into gene tree reconciliations. As we demonstrate in our empirical example (Figure 3), the optimal reconciliations from our likelihood approach can differ from the parsimony reconciliations, and we suggest they may be more accurate when genes have high rates of duplication and loss [9,10].

Our approach also is fundamentally distinct from previously described models based on the birth-death process [21-23]. Not only can our approach incorporate a greater range of possible distributions for the duplication and loss process, in general, while the birth-death models infer a branching process for the gene trees, our modeling approach directly aggregates duplications on the edges of a species tree. Also, unlike other modeling approaches [22,23], we assume that the gene tree topology is fixed; we do not incorporate nucleotide substitution models or attempt to simultaneously infer the gene tree topology and reconciliation. Thus, although our approach may be more easily misled by gene tree error, our approach is computationally much less complex in practice.

The models and algorithms described in this paper provide the foundation for a rigorous statistical framework to test assumptions about the rates and patterns of gene duplication and loss. In fact, a key feature of our algorithmic approach is that it provides a generic modeling framework in which to compare the likelihood of different distributions of gene duplication and loss throughout evolutionary history. The main disadvantage of a likelihood-based approach compared to parsimony is the computational cost associated with the likelihood function. However, our analyses of simulated and empirical data sets demonstrate that our likelihood approach is computationally feasible even for trees with hundreds of taxa. We note that all models are imperfect representations of actual processes, and furthermore, it is difficult to predict the best model for any specific problem or data set. While the fit of different models will depend on the complex, and largely unknown, selective constraints guiding a gene’s evolution, the utility of a model is also a function of its statistical power and robustness to violations of its assumptions. Much future work, involving both simulation experiments and analyses of empirical data sets, is needed to fully characterize and compare the performance of these different modeling approaches. Still, the availability of new modeling options will only enrich the study of gene family evolution by providing new opportunities for model comparison studies.

Directions for future research include: (i) allowing soft multifurcations in gene and species trees, (ii) improving the performance of the prototype program in case of hard instances and (iii) characterizing the performance of this approach through gene tree simulations.

## Authors’ contributions

PG and OE were responsible for developing the solution. PG was developing the code and running the experiments. PG and JGB performed the experimental evaluation and the analysis of the results. All authors contributed to the writing of the paper, read and approved the final manuscript.

## Competing interests

The authors declare that they have no competing interests.
